# Expected Impacts of COVID-19: Considering Resource-Limited Countries and Vulnerable Population

**DOI:** 10.3389/fpubh.2021.614789

**Published:** 2021-05-05

**Authors:** Tigist Gashaw, Bisrat Hagos, Mekonnen Sisay

**Affiliations:** ^1^Department of Pharmacology and Toxicology, School of Pharmacy, College of Health and Medical Sciences, Haramaya University, Harar, Ethiopia; ^2^Department of Social Pharmacy, School of Pharmacy, College of Health and Medical Sciences, Haramaya University, Harar, Ethiopia

**Keywords:** coronavirus, pandemic, impact, comorbidity, vulnerable

## Abstract

Coronavirus disease in 2019 emerged in Wuhan, Hubei Province, China, in December 2019. After a month, it was declared a global threat to public health. The effects of the pandemic could be socio-economic, undermining the health system and risking livelihoods. Vulnerability to this infection has been associated with underlying comorbidities such as hypertension, diabetes, coronary heart disease, chronic respiratory diseases, cancer, and compromised immune systems. Co-morbidity has been common to the elderly, the disabled, and the homeless. In addition, more severe coronavirus disease outcomes have been reported in older males than females. Nonetheless, multiple variables are related to the concept of cultural gender that should be taken into account as women in more affected sectors are economically disadvantageous and over-represented. Similarly, although children are not the face of this pandemic, calamity has a profound effect on their welfare, especially for those living in poor and inconvenient situations. Moreover, the economic influence could be profound and universal when viewed through a migration lens as it is exacerbating xenophobic and discriminatory treatment. Protection measures to mitigate the outbreak of a pandemic, such as social distancing, may reduce social support for certain categories relied on for their day-to-day activities. The mental health of people would definitely be affected by the additional psychosocial burden of the pandemic, particularly in vulnerable groups. Integrated approaches are therefore mandatory to assist these groups and contain the pandemic.

## Introduction

Coronavirus disease in 2019 emerged in Wuhan, Hubei Province, China, in December 2019. After 1 month, as it became highly infectious and spread rapidly to most countries, the World Health Organization (WHO) declared it a global public health threat (1, 2). Looking at the case burden, the United States of America is on the frontline, and African countries have a lower rate of accounting (3). However, researchers warned that cases could go undetected in countries with weaker healthcare systems, such as some in Southeast Asia and Africa, which could be quickly stunned by the local epidemic (4). Although it is difficult to predict precisely the consequences of the COVID-19 pandemic, some of the predisposing factors may be elucidated on the basis of the experiences of previous pandemics and the already COVID-19-related crises that have been magnified from time to time, mostly in poor countries. Hence, this perspective aims to elucidate the expected impact of COVID-19 in the context of resource-limited countries and vulnerable populations.

## Impacts of COVID-19

### Socioeconomic

Concerns arose about some countries with a modest risk of developing the virus, but whose delicate health systems, economic position, or unstable political situation are increasing vulnerability to the impact of the COVID-19 pandemic, such as Nigeria, Ethiopia, Sudan, Angola, Tanzania, Ghana and Kenya (4). The impact on food security could be rapid and catastrophic, especially for people living in food crises. Potential impacts on food security are mainly linked to movement restrictions, as more than 75% of them rely on rural farming as a means of support. This may lead to migration and displacement in the search for assistance (5). In addition, the lockdown prevents 300 million school children in Africa from accessing school meals on which their nutrition depends. The situation is even more distressing in East Africa, where coronavirus is hindering efforts to combat one of the largest locust swarms in recent times (6).

The household was also considered a key context in the analysis of the transmission of COVID-19. The mediocre number of families with a resident of more than 65 years of age was significantly higher in low-income countries compared to middle-and high-income countries, increasing the possibility of spread in general and among vulnerable age groups in particular (7).

The threat from COVID-19 has already changed to a drop in the economic and labor market (8). It was suggested that a one percent decline in global economic growth would be interpreted as more than 22 million people prone to poverty (9). Considering Africa as an example, the high dependence of the continent's foreign-referred economies predicts a negative spin-off and is estimated at a 1.5-point average decline in economic growth in 2020. Moreover, the continent is lagging in the transformation of locally available raw materials to respond to the potential high demand for goods and amenities on the internal and global markets (10). In relation, street vending (street food sales and wet market engagement) is an important source of income for many poorer segments of society in urban areas in developing countries. Most people join this business sector to be self-reliant, to support their families, and to lack of opportunities to engage in the formal sector (11, 12). Approaches to mitigate the effects of virus-like movement restrictions or physical distances may therefore challenge the livelihoods of these individuals, resulting in food insecurity and malnutrition (5, 13).

Another negative impact expected from the COVID-19 pandemic could be explained by the social and political instability associated with the suspension of elections, which could trigger many political actors from opposing parity with the ruling government, generating multidimensional tensions (14). As a result, these effects further fuel societal difficulties in accessing survival requirements and lead to reduced integrity (15–17).

### Health System

The crisis of COVID-19 is spreading to already existing poor health systems and the fragile economy of resource-limited countries (18). Lower ratios of hospital beds, intensive care units, and health professionals to their population, as well as dependence on imported medicinal and pharmaceutical products, could be identified as a problem (19, 20). The practice in different nations has highlighted the deep stress that a COVID-19 epidemic poses on countrywide health systems, particularly in increasing the need for intensive care facilities everywhere (21). This has a potential impact on resource-scarce areas where the availability and quality of these resources are significantly trivial (22, 23). Besides, though there was a prediction of a lower incidence of severe COVID-19 outcome in lower-income countries due to the younger average age, it was likely to be offset by an absence of a sufficient health care system (7, 24). Besides, many of them are behind the ability of tests to randomly detect cases that fit their population size.

### Vulnerable Population

#### Older People With Underlying Co-morbidities

The crises brought by the COVID-19 pandemic can have an unequal impact on certain sections of the population (25). Various studies are trying to advance the basic determinants of the severity of the infection of COVID-19. Primary factors include age, with the incidence of underlying co-morbidities such as hypertension, diabetes, coronary heart disease, chronic respiratory diseases, cancer, and compromised immune systems commonly used to exacerbate symptoms (26–29). Besides, the deleterious emotional impact of the outbreak may interfere with the clinical outcomes of patients with chronic conditions such as cardiovascular disease and mental illness, whose progress and management are related to anxiety and stress (30). Infection control measures, in particular movement restrictions, will also result in a significant decrease in physical activity and an increase in unhealthful lifestyles, worsening of clinical symptoms of most chronic conditions (31). Up-scaling strategies to ensure adherence to drugs must be another area of concern. Patients who remain in follow-up care are more likely to follow their treatment and experience improved health outcomes (32, 33) than those who are not (34) which will likely happen during the COVID-19 pandemic. It is also evident that if the burden of the pandemic is escalating, it will be challenging to treat these patients as lessons from the Ebola pandemic showed a decline in primary medical consultations in more than half of cases (35).

Thus, in order to maximize adherence to drugs, healthcare providers can disseminate health education and drug information to patients by implementing new practices via phone calls, SMS text messaging, and social media platforms. For those who cannot access these technologies, carrier services could be organized for household distributions of essential medicines and prescription refills by strict implementation of all anti-COVID-19 safety protocols (36). In addition, enhancing the role of community pharmacists in managing chronic conditions and maintaining drug adherence during this pandemic is a critical area in alleviating the burden on already stressed health systems (37). Thus, focusing on the virus is justified, a balanced approach to avoiding unnecessary morbidity and mortality is imperative (38). Integrated strategies from local health units, community organizations, and healthcare systems are needed to support and contain these vulnerable groups in the event of a pandemic (33, 39–41).

#### Women

##### Biological Implications of Female Sex on COVID-19 Pathogenesis

Various studies provide evidence that suggests the severity and clinical outcome of COVID-19 infection are more lethal in infected males than females, although sex-disaggregated data in several European countries (42, 43) and China (44–46) showed similar prevalence among infected males.

Medical assumptions have been made that the biological effect of the female sex is protective against COVID-19 pathogenesis.

In females, estrogen is responsible for increasing the expression and activity of endothelial nitric oxide synthase (eNOS) and nitric oxide (NO) in the systemic vasculature of females (47). Physiological NO signaling is the main determinant of endothelial function and vascular health. It is the main regulator of the vascular smooth muscle and has anti-inflammatory, antioxidant, and anti-thrombotic activity as well as the defense of the vascular system against vasoactive contractors such as angiotensin II (48). Females are therefore naturally at a lower risk of cardiovascular disease and persistent endothelial dysfunction, which is a major factor in increasing susceptibility to severe acute coronavirus 2 (SARS-CoV-2) infection due to increased levels of angiotensin-converting enzyme 2 (ACE2) (SARS-Cov-2 receptor) exposure to vascular and cardiac pericytes (49, 50).

Also, females are identified to produce more innate and adaptive immune responses than males, which may help to clear the virus more quickly (51). The number and activity of innate immune cells, including monocytes, macrophages, dendritic cells, and inflammatory immune responses, are increased in females compared to males (52). And the adaptive immune responses also reveal higher humoral and cell-mediated immune responses to antigenic stimulation, vaccination, and infection than do males (51). Besides, females' immunoglobulin (53) along with antibody responses is steadily superior (54).

##### Sociocultural Attributes of Gender on COVID-19 and Vice Versa

Gender is defined as the social and cultural norms, roles, attributes, and behaviors that a society considers appropriate for men and women or boys and girls (55–57). Data indicate an association between comorbidities and severity of COVID-19 (58, 59). Women are generally at a lower risk of developing morbidity than men (60), except for older age groups. Gender differences in risk behaviors have been reported to play a pivotal role in determining the death rate of non-communicable diseases (61). Persistently higher smoking and risky drinking behaviors are associated with men than women worldwide, leading to the development of comorbidities. Moreover, delayed health-seeking behavior and lower rates of hand-washing patterns have been reflected in men and these are also imperative measures in the era of COVID-19 (42).

However, a number of factors are related to gender definition and should be analyzed before a conclusion is made. Regardless of one's socio-economic class, there are systematic gender differences in material well-being, although the degree of inequality varies across countries and over time. As a result, most societies have gender disparities, with males generally holding higher positions in social, economic, and political hierarchies (62). Gender disparity is not merely perpetuated by unequal access to and ownership of material resources. Gender roles and expectations preserve gender identities and restrict the actions of women and men in ways that contribute to discrimination (63, 64).

In developing countries, where poverty and social norms create even worse unequal treatment for girls and women, the consequences are likely to be even more serious. Women are prevented from entering the formal sector by cultural traditions, religious seclusion, and illiteracy, as well as a greater devotion to family obligations (65, 66). The part of any economy that is neither taxed nor controlled by any kind of government is known as the informal economy (informal sector or gray economy) (67) which offers vital economic opportunities for the vulnerable like women who make up the majority of the informal sector (68, 69). Home-based workers (homemade) and street vendors are two major occupations in this sector (70). However, the majority of employees in the gray economy, lack stable jobs, benefits, welfare, or representation (65, 71, 72).

Moreover, women are highly engaged in the more affected areas of the COVID-19 pandemic (e.g., nurses, janitors) and account for a large proportion of careers (73). Mostly, they do not have access to social protection and are forced to accept an imbalanced burden in the care economy due to the closure of schools or care systems (74, 75). Most of them will therefore be engaged in informal sectors predisposed to increased risks of gender-based violence. Due to traditional influences in developing countries, women may not be in a position to make decisions that hinder the ability to obtain information on outbreaks and the availability of services such as sexual and reproductive health services, including family planning (76) resulting in increased unplanned pregnancy.

The burden of the pandemic on women's health is not only limited to reproductive health but also their emotional attachment and concern for their family well-being, along with a decline in social interaction that could lead to mental health complications (77–80).

The influence of gender variables on disease manifestations and outcomes should therefore be thoroughly assessed in every local context, and the impact of these variables should be incorporated into policies and actions that may be implemented at different levels. These measures particularly benefit underprivileged populations and resource-poor communities like us, where women are particularly vulnerable.

#### Pregnancy

Based on available information, the risk of COVID-19 during pregnancy seems to have the same as adults and non-pregnant. However, caution must be taken as this group of people has a history of higher risk of severe illness when infected with viral respiratory infections, such as influenza. The changes brought by pregnancy may exacerbate the risk of these infections. It is therefore advisable to take maximum care of this special category to protect themselves from illness whenever possible (28, 81). A further area of concern will be the protection measures for the pandemic, such as social distancing, which may have a bad attribute in isolation and reduced social support, which may lead to mental distress, involving the COVID-19 related policy guidelines, which should categorically include maternal mental health and well-being as a priority area (77, 82, 83).

#### Children

Children are generally at less risk of more serious diseases and complications compared to adults. However, indirect effects raise questions about their welfare, especially in the poorest countries, and vulnerable situations (84). Rising malnutrition is expected after school closure across the majority of countries where school meals have been a reliable source of daily nutrition (85, 86). Increased vulnerability is responsible for external and internal displacement, arrest, and conditions of conflict. A rise in mortality rates associated with decreases in maternal and child health care coverage has also been estimated (87).

In addition, a greater psychological issue may be enforced by movement limitations compared to the physical sufferings caused by the virus. Static and recurrent lifestyle styles are likely to disrupt the behaviors of adolescents, potentially stimulating suffering, intolerance, monotony, agitation, and multi-neuropsychiatric indications. They are also predisposed to domestic violence, abuse, and uncontrolled virtual content (88).

It is expected that policymakers will respond by extending social security programs to cover the most vulnerable children by prioritizing child-centered services to alleviate the effects on this particular population. Besides, it is important to provide meaningful support to parents and caregivers on how to interact with children, address mental health concerns, and build tools to support learning.

#### Homeless Population

Living without a home is a major predisposing factor for an unhealthy lifestyle (89). Homeless people often have many complex health issues (the co-existence of physical, mental, and addiction problems) and face several barriers to accessing health care as well as public health information (90, 91). In the current COVID-19 pandemic, due to their weakened immune systems, inadequate nutrition and hygiene, and long-term residence in overcrowded shelters, they are at higher risk of contracting infectious diseases and mortality rates with a limited percentage of identification and care (92) implicating that they are a highly vulnerable community (93, 94). Moreover, being without a permanent residence address makes it difficult to track and inhibit the spread and treat those in need (92, 95). This could lead to less inclusion of the community in search of knowledge during pandemics and thus overlooked by the subsequent policies (96) missing the fact that the safety of these individuals is an important component of handling the broader disaster of public health.

#### Informal and Migrant Workers

The disastrous impact on the economy brought by COVID-19 could be stretched, intense, and worldwide when observed from migrants' prospects. All measures related to movement restrictions resulted in global economic actions to close to cessation (97, 98) fueling the already exiting unemployment and underemployment in young's and informal workers as witnessed during the global financial crisis (99). Migrant workers incline to be susceptible to the loss of jobs and earnings in an economic calamity in their host country higher than inborn employees (100). Numerous migrants have been stuck owing to the postponement of transference and lockdown in labor camps increasing the risk of infection among them (75). However, host countries have permitted visa extensions and momentary amnesty, as well as delayed migrants' spontaneous departure (97). In 2020, remittance flows to developing countries are expected to decline in 2019 by about 20 percent. This may be due to worsening famine and reducing the access of families to vital health services since it is a source of economic sustenance (101, 102). The results of the studies also showed a positive significant correlation between per capita gross national income and net migration and the occurrence of total cases of COVID-19 and new cases regularly (103). In evaluating the total and new cases of COVID, the scale of net migration proved to be a possible factor and a positive factor. Xenophobic and racist treatment of migrants may also be aggravated by crises (104).

These activities call for greater vigilance and action by administrators to resolve the problems of migrants by including them in health care, social initiatives, and shielding them from discrimination. Studies also show that the pandemic affects mental health, with extreme symptoms of depression and anxiety in migrant workers (98, 105). Therefore, mental health is a critical aspect that needs to be addressed by generating awareness and psychological support.

#### People Living With Disability

In terms of greater health needs, individuals living with disabilities (PLWD), including physical, emotional, intellectual, or sensory disabilities, are excluded from accessing health care, resulting in poorer outcomes and disparities (76). To aggravate these discriminations, COVID-19 intimidates primarily in resources restricted to countries where 80% of PLWD reside and with insufficient capacity to respond to COVID-19 (106, 107). Many of these categories of people are challenged by existing chronic conditions—such as heart disease, diabetes, and obesity—that predispose them to developing and distressing COVID-19 difficulties (108, 109). Studies point out that although some optimistic action has been taken, the needs of people with disabilities have not been fully addressed. Many nations' response to COVID-19 included disability-comprehensive recommendations, but without ensuring implementation (110). So, readiness and response preparation must be inclusive and easy to get to PLWD in tackling key barriers (111). It is also important to disaggregate information related to disability groups during the pandemic for both those who have beaten COVID-19 and those who have surrendered to the disease. This will leave lessons and experience in the development and implementation of fitting interventions for imminent public health crises in these vulnerable populations (112).

## Integrated Strategic Approaches Against the COVID-19 Pandemic and Lesson Learned in Combating the Crises Mirroring Ways to Safeguard the Future

### Mitigation Strategies

There is a serious need to discover alternative public health prevention methods to control the spread in the absence of treatment for the COVID-19 virus. By realizing that there is no one-size-fits-all solution to managing the outbreak, all countries should increase their level of preparedness and response to various public health scenarios, since each country has its specific contextual strengths and flaws to account for the impact (113). A cohesive strategy against the COVID-19 epidemic was struggling to be developed by many countries with poorer health systems, including lockdown, physical distance interventions, wearing a facemask, and successful case finding (testing and isolation, contact tracing, and quarantine) (114).

Using the modeling approach, limited resource settings were studied to be best served by a combination of early and aggressive case findings and ongoing physical distancing (which alone might prevent the pandemic from 60–95%, if timely and effectively implemented) measures to control the epidemic. But a lockdown may be helpful until combination interventions can be put in place as it is unlikely to reduce annual mortality or healthcare demand (115). It was also concluded that most countries benefited from early intervention (114) along with community involvement, such as active involvement of religious institutions and mobilizing young people to increase public awareness and community-level case detection through the health extension program (116). Moreover, the role of social media has been significant in promoting public health responses to both preparedness and outbreak control by teaming up with the government to deliver clear, simple, and consistent messages (117).

### Capacities of Laboratory Facilities Should Be Enhanced

Despite the rapid increase in the number of COVID-19 cases reported globally, limited molecular laboratory capacity is evident in resource-constraint settings for case diagnosis and overall disease management. As the Ethiopian Public Health Institute (EPHI) shared the experience of the rapid establishment of one of its COVID-19 research laboratories from locally available resources, the optimal use of existing resources and the repurposing of other sources for human medical treatment could be considered as a preferable approach to the management of restricted facilities (118, 119).

### Healthcare Systems Values That Need to Be Maximized

Health is a prerequisite for living a “good life.” to achieve other goals that people want to pursue in life. The universal healthcare system should therefore seek to provide the population with a comprehensive concept of health, including the participation of patients in the delivery of care, the best possible use of available resources, the equitable distribution of resources across all patient groups, and the contribution of healthcare to social participation and connectivity (120). In addition, as a strategic objective, the health system should value dynamic leadership driven by transparency and enhancement of value (121). These principles of the health care system will result in a predictive, personalized, preventive, participatory, and psycho-cognitive medicine (122) that is essential in the Pandemic Control Intervention of COVID-19.

### Attentions of Internationally Funding Organizations

The goals of the Global Humanitarian Response Plan are to maximize coherence and provide an efficient and effective humanitarian response that, through active community participation, will be incorporated into all of the nations' current intervention response plans. The guiding principle for these humanitarian activities is a people-centered approach and inclusiveness, especially for the most vulnerable, stigmatized, hard-to-reach, displaced and mobile populations marginalized in national plans. Also, a tailored response will be given to the needs of different age groups (children, the elderly) as well as to gender equality, in particular, to address the unique needs of women and girls, the risks and roles of providing care, increased exposure to movement-restricted gender-based violence and a large number of female frontline health workers in this pandemic response (119, 123–125).

### Vaccine Policy

Implementing national-level vaccination programs involves long-term investment, which can be a significant financial burden, particularly in resource-limited settings (126). Reports revealed that, before the start of marketing, high-income countries had made deals to buy COVID-19 vaccines but left the rest of the world with unclear access. The uncertainty of global access to COVID-19 vaccines is due not only to ongoing clinical trials but also to the inability of governments and vaccine manufacturers to be more transparent and accountable for these arrangements, from equal pricing to fair allocation (127). This will also present threats of corruption that could jeopardize critical public health objectives. These concerns include the provision of counterfeit and substandard vaccines, theft, nepotism, racism, and corrupt procurement processes. Responsible authorities are indeed required to act to prevent corruption in the production, allocation, and distribution of vaccines in the supply chain (128–130).

In order to help solve some of the related problems, SAGE (The Strategic Advisory Group of Experts on Immunization) proposes a prioritization roadmap for COVID-19 vaccines that considers priority groups for vaccine versions based on epidemiological settings, vaccine supply scenarios, and the context of the overall public health strategy for each epidemiological setting (131–133). The WHO has also established the principle that vaccines for COVID-19 must be a global public good. The key objective of COVID-19 vaccines is to contribute significantly to the equitable protection and promotion of human well-being, based on reciprocity and legitimacy for all people around the world, at the global and national level (130, 133, 134). Besides, focus should be put on older adults, pregnant and lactating mothers in clarifying gender-based biological disparities in the efforts of vaccine production clinical trials to ensure safety and efficacy (135, 136).

Currently, three vaccines are authorized and recommended to prevent COVID-19: Pfizer-BioNTech, Moderna, and Janssen. As of February 2021, large-scale (Phase 3) clinical trials are in progress or being planned for two COVID-19 vaccines in the United States: AstraZeneca COVID-19 vaccine and Novavax COVID-19 vaccine. Forecasting vaccine and logistics needs, evaluating available storage space, defining surge capacity, developing a delivery plan, reinforcing supply and stock management, implementing a vaccine traceability system, and planning for vaccine protection and concerned workers are among the main approaches proposed for vaccine deployment. Furthermore, many low- and middle-income countries will face many challenges when it comes to COVID-19 vaccines with an ultracold chain (UCC) (−60°C to −90°C degrees °C) profile, including a lack of current UCC equipment in health/immunization systems, including phase change material (PCM) and facilities to manufacture dry ice; high investment costs due to the time-limited existence of the need for UCC capability—many countries will aim to move to vaccines that can be stored at +2°C to +8°C; and difficult handling and distribution requirements, especially where UCC products have limited shelf lives (e.g., 7 days) when stored at +2 to +8 degrees Celsius. As a result, countries in need of UCC can consider feasible options such as utilizing existing internal and/or external resources (137–142).

### Lesson Learned in Combating the Pandemic, Outlook, and Preparedness to Safeguard the Future

To plan for potential outbreaks, countries should draw on lessons from COVID-19. This pandemic has restated the prominence of the saying “prevention is better than cure” and has trained mankind mentally to fight and battle this pandemic. It also revealed weak points about how we're thinking about health and preparing for the disease. It has also created an ability to enhance our services and health care systems and learn how to be better positioned for the next emergency (116). On the other hand, by taking advantage of the opportunity provided by the current pandemic, different settings have benefited from the establishment of an additional standard molecular laboratory, trained staff as well as the attribution of human and material resources that could last beyond the COVID-19 era (118).

A further limitation noted in the health sector was the lack of a harmonized approach to data governance and global standards for terminology and sharing of health data. It must be improved so that it can provide usable and accessible national and global data for surveillance and emergency response without neglecting confidentiality (143).

The fundamental link between human factors, development, and technology was also exposed to the pandemic of COVID-19. Healthcare programs are structured to ensure health security and progress in the health sector, but they are too often under-funded, under-employed, stressed, and placed at the bottom of the political agenda. In addition to stemming this outbreak, efforts should therefore be made to develop comprehensive measures to prevent future outbreaks (116) by maintaining biosecurity, through the One Health Approach (to achieve the best health outcomes for humans, animals, and the environment) and by providing information on recent developments (144). Ultimately, the magnitude of the crisis will be determined by our mutual ability to recognize and respond to these interdependencies through integrated strategies to control COVID-19, including vaccination, molecular and serological diagnosis, hygiene and WaSH (water, sanitation, and hygiene) interventions, and low-cost therapeutics (145–152). These aspirations should be at the heart of the health inclinations of the global community in the post-COVID-19 era (153). Besides, psychosocial risk mitigation should be a key focus, and policymakers can give priority to working on a simple yet cost-effective organizational exercise to enhance disaster coping mechanisms (149, 150).

Further research focusing on the screening, identification, isolation, and characterization of coronavirus (CoV) in wildlife species, particularly in bats, should be continued through both *in vitro* and *in vivo* methods, as emerging patterns suggest future outbreaks of CoV due to climate change and ecological conditions that may be associated with human-animal contact (154–156). Moreover, post-marketing surveillance should be considered for the tracking and evaluation of approved vaccines.

## Conclusion

The impacts of COVID-19 have been revealed multidimensionality including socioeconomic, weakening the health system, and increasing the vulnerability of some segment of the population. Developing appropriate strategic measures, keeping and upscaling food security interventions, and protecting the livings of the most vulnerable people such as those with underlying co-morbidities, children, homeless, women, pregnant, migrants, and people with disabilities need to be top priority. Supporting cities and local managements in their actions to mitigate the calamity by sharing and increasing involvement is also central. Methods to evaluate the effectiveness of current health interventions combined with a high level of testing proportional to countries population size should also be accentuated. Moreover, the interrelationship between characteristics of high-risk populations for impact of COVID-19 and health outcomes and prompt consideration of these variables in both research, clinical practice, and vaccine development should be acknowledged. Moreover, COVID-19 vaccines must be a global public good that is aimed to contribute significantly to the equitable protection and promotion of human well-being at the global and national level based on reciprocity and legitimacy. When the crisis ends, countries should draw lessons from COVID-19 to prepare for future outbreaks.

## Data Availability Statement

The original contributions generated for this study are included in the article/supplementary material, further inquiries can be directed to the corresponding author/s.

## Author Contributions

TG drafted the document and prepared the final version for publication. All authors had substantial contributions to the document, read and approved the final version of the manuscript.

## Conflict of Interest

The authors declare that the perspective document was conducted in the absence of any commercial or financial relationships that could be construed as a potential conflict of interest.
